# A TiO_2_ Coated Carbon Aerogel Derived from Bamboo Pulp Fibers for Enhanced Visible Light Photo-Catalytic Degradation of Methylene Blue

**DOI:** 10.3390/nano11010239

**Published:** 2021-01-18

**Authors:** Jian Zhang, Wei Yuan, Tian Xia, Chenghong Ao, Jiangqi Zhao, Bingxue Huang, Qunhao Wang, Wei Zhang, Canhui Lu

**Affiliations:** 1 State Key Laboratory of Polymer Materials Engineering, Polymer Research Institute at Sichuan University, Chengdu 610065, China; pirlo3721@126.com (J.Z.); Union_sing@126.com (W.Y.); xiatian0621scu@163.com (T.X.); chenghongaocd@163.com (C.A.); scu_jqz@163.com (J.Z.); hbx15756376063@126.com (B.H.); 17839918692@163.com (Q.W.); 2 Advanced Polymer Materials Research Center of Sichuan University, Shishi 362700, China

**Keywords:** cellulose, carbon aerogel, titanium dioxide, photo-catalytic degradation, methylene blue

## Abstract

Carbon aerogels (CA) derived from bamboo cellulose fibers were coupled with TiO_2_ to form CA/TiO_2_ hybrids, which exhibited extraordinary performance on the photo-catalytic degradation of methylene blue (MB). The structure and morphology of CA/TiO_2_ were characterized by field emission scanning electron microscopy, Fourier transform infrared spectroscopy, X-ray photoelectron spectroscopy, X-ray diffraction, and Raman spectrum. The CA displayed a highly porous and interconnected three-dimensional framework structure, while introducing the catalytic active sites of TiO_2_ onto the aerogel scaffold could remarkably enhance its photo-catalytic activity. The adsorption and photo-catalytic degradation of MB by the CA/TiO_2_ hybrid were investigated. The maximum adsorption capacity of CA/TiO_2_ for MB was 18.5 mg/g, which outperformed many similar materials reported in the literature. In addition, compared with other photo-catalysts, the present CA/TiO_2_ demonstrated superior photo-catalytic performance. Almost 85% of MB in 50 mL solution with a MB concentration of 10 mg/L could be effectively degraded by 15 mg CA/TiO_2_ in 300 min.

## 1. Introduction

The vast application of dyes in the light industry, especially in textile, leather, paper, plastics, and so on, to obtain colorful products has become a recurring phenomenon. However, residue dyes in effluents raise huge environmental problems which threaten ecosystems, including those on which we as human beings rely. For example, as one of the most frequently used dyes, the discharge of methylene blue (MB) is a major concern due to its adverse impacts on health [1]. Inhaling MB can cause accelerated or difficult breathing. If orally administrated, the affected person may show some strong clinical responses such as burning sensations, mental confusion, vomiting, nausea, and methemoglobinemia [2,3,4]. Hence, the development of eco-friendly and more efficient strategies for the removal of MB in wastewater is of great significance for the aquatic environment.

So far, various methods have been applied to tackle MB pollution in wastewater, such as sonochemical degradation [5], cation exchange [6], electrochemical degradation [7], micellar-enhanced ultrafiltration [8], Fenton-biological treatment [9], and adsorption/precipitation [10]. Among them, photo-catalysis shows promise to settle this problem. As is well known, photo-catalysis can fully degrade organic dyes into small molecules without any secondary pollution and is free from the subsequent separation process [11,12,13]. Titanium dioxide (TiO_2_) is one of the most popular photo-catalysts, owing to its excellent photo-catalytic performance, low cost, superior stability, and non-toxicity.

Meanwhile, doping with nonmetals (C, N, S) has been proven to be able to produce photo-catalysts with higher efficiencies under visible light [14,15,16,17,18,19]. Specifically, its combination with carbon materials can largely improve the visible light response and photo-catalytic activity [20,21,22,23,24,25,26,27,28,29,30,31,32,33,34]. For example, Esther et al. prepared a carbon xerogel–TiO_2_ composite through a sol–gel process followed by pyrolysis using resorcinol and formaldehyde as the carbon sources [35]. The micro-morphology analysis indicated that the carbon sphere was coated by TiO_2_. The interaction between carbon and TiO_2_, as well as the oxygen vacancies caused by carbonization, could narrow the band gap of TiO_2_ and consequently improve the visible light response of the composite.

Nonetheless, most photo-catalysts reported previously showed lots of defects and boundaries caused by the aggregation of the carbon matrix. This will inevitably result in the recombination of photo-generated electrons and holes and decrease the photo-catalytic efficiency. Carbon aerogels, a novel species of porous carbon material, have shown promise in various fields [36]. Nonetheless, carbon aerogels are usually derived from synthetic organic compounds. This not only causes fast consumption of the limited non-renewable resources but also brings about secondary pollution to the environment after their service life. Moreover, there are only limited studies focusing on the application of carbon aerogels in photo-catalysis.

Herein, we present a carbon/TiO_2_ hybrid aerogel (CA/TiO_2_) using bamboo pulp fibers as the carbon source for the photo-catalytic degradation of MB under visible light. Bamboo is an abundant natural resource in Southwest China. Compared with wood, it only takes several months to grow. In addition, it has a high content of cellulose, with many distinguishing features such as high strength, high flexibility, and low density. Additionally, our previous study suggested that bamboo pulp fibers could be used as a promising precursor material for the preparation of carbon aerogels with ultra-lightweight and high porosity features [37]. Meanwhile, TiO_2_ was hydrothermally anchored on the surface of fibers and distributed homogeneously along the fibers. The obtained hybrid aerogel was still highly porous with an interconnected three-dimensional framework. This structure could effectively suppress the generation of boundaries and defects and hence decrease the probability of recombination between photo-generated electrons and holes, leading to improved photo-catalytic activity under visible light. In the meantime, the aerogel structure also favored the adsorption of MB as it promoted dye diffusion and provided a higher specific surface area.

## 2. Materials and Methods

### 2.1. Materials

The bamboo pulp with a solid content of 25 wt% was supplied by Yongfeng Paper Co., Ltd., Muchuan, China. Tetrabutyl titanate (Ti(OBu)_4_), acetic acid, and ethanol were of analytical grade and supplied by Kelong Chemical Reagent Co., Ltd., Chengdu, China.

### 2.2. Synthesis of CA/TiO_2_

The CA/TiO_2_ was fabricated by a hydrothermal method using bamboo cellulose and Ti(OBu)_4_ as the carbon source and titanium dioxide precursor, respectively. In a typical process, bamboo pulp with a determined mass was dispersed in 25 mL deionized water under magnetic stirring to form a homogeneous suspension with a concentration of 1 wt% (solution A). Meanwhile, 1 mL Ti(OBu)_4_ was added to 5 mL ethanol containing 2.5 mL acetic acid under stirring to form a light yellow solution (solution B). Next, solution B was added into solution A and stirred for 2 h. The mixture was then transferred into a Teflon-lined stainless-steel autoclave and kept at 150 °C for 6 h. After that, the precipitation was centrifuged while it cooled down to room temperature. The obtained powder was re-dispersed into deionized water. Then, the suspension was freeze-dried at −40 °C for 48 h to obtain the cellulose/TiO_2_ hybrid aerogel.

In order to produce CA/TiO_2_, the cellulose/TiO_2_ hybrid aerogel was subjected to high-temperature carbonization in a tube furnace (OTF1200X, Kejing Materials Technology Co., Ltd., Shenzhen, China). The sample was heated to 240 °C at a heating rate of 2 °C/min and kept at this temperature for 1 h. Then, the temperature was increased to 400 °C at a rate of 2 °C/min and kept for 1 h. After that, the temperature was raised to 600 °C at 5 °C/min, followed by an isothermal process for 1 h, and then further heated up to 800 °C at a rate of 5 °C/min and kept for another 2 h. Finally, the sample was cooled down to the ambient temperature naturally. All the pyrolysis processes were conducted in a nitrogen atmosphere. For comparison, a pure carbon aerogel was also prepared in a similar way and denoted as CA.

### 2.3. Characterization

The micromorphology of CA/TiO_2_ was observed through field emission scanning electron microscopy (FE-SEM, JEOL JSM-7500F, Tokyo, Japan). The relative contents of the elements were analyzed via energy dispersive X-ray spectroscopy (EDX). Fourier transform infrared spectroscopy (FTIR) and X-ray photoelectron spectroscopy (XPS) were used to characterize the chemical composition of CA/TiO_2_. FTIR spectra were acquired from 400 to 4000 cm^−1^ at a resolution of 4 cm^−1^ using a FTIR spectrometer (NICOLET 6700, Thermo Fisher Scientific, Waltham, Massachusetts, USA). XPS spectra were recorded on a XPS spectrometer (ESCALAB 250Xi, Thermo Scientific, Waltham, Massachusetts, USA) with an Al Kα X-ray source (1486.8 eV). The crystalline structures were observed through X-ray diffraction (XRD, D8 Advance, Bruker, Karlsruhe, German) using Cu Kα radiation at a wavelength (λ) of 1.541 Å with 2θ angle from 10 to 70°. Raman spectra were obtained on a Raman microspectrometer (STA 6000, Perkin Elmer, Waltham, Massachusetts, USA) with an excitation wavelength of 532 nm. Optical absorption behavior was characterized by a double-beam UV–vis spectrophotometer (UV-3600, Mapada Instruments Co., Shanghai, China) equipped with a Praying Mantis diffuse reflectance accessory (DRS); BaSO_4_ was used as a reference.

### 2.4. The Adsorption Performance for MB

A certain amount of CA/TiO_2_ was immersed into 20 mL solution with the MB concentration varying from 1 to 20 mg/L under dark conditions and shaken for 2 days at ambient temperature. The residual concentration of MB in the solution was calculated by the absorbance of the measured solution according to the standard curve of the MB. When the adsorption equilibrium was reached, the concentration of the remaining MB was evaluated. The concentration of the MB was detected by a UV–vis spectrophotometer at 664 nm. The adsorption capacity of CA/TiO_2_ for MB was calculated by Equation (1):

(1) $$q_{e}=\frac{V\left(c_{0}-c_{e}\right)}{m}$$

where *q_e_* (mg/g) is the adsorption capacity for MB, *C*_0_ and *C_e_* (mg/L) are the concentrations of MB before and after adsorption, respectively, *m* (g) is the mass of CA/TiO_2_, and V (L) is the volume of MB solution.

### 2.5. Photo-Catalytic Activity Test

The photo-catalytic activity of CA/TiO_2_ was monitored via the change of MB concentration under the illumination of an ordinary incandescent lamp (200 W, Shuangyi Lighting Electric Appliance Co., Ltd., Shenzhen, China). Firstly, 15 mg CA/TiO_2_ was added into 50 mL MB solution with a concentration of 10 mg/L for MB adsorption under dark conditions. When the adsorption–desorption process reached equilibrium, the mixture was transferred to the irradiation region of visible light. Equal aliquots of solution were withdrawn from the system every 30 min and measured at 664 nm by a UV–vis spectrophotometer to estimate the concentration of the remaining MB. The photo-catalytic performance was evaluated by the ratio of *C*/*C*_0_, where *C* is the initial concentration of MB and *C*_0_ is the concentration of MB at time t. The experiment was conducted for three times and the average values were presented. In addition, the experimental data of photo-catalytic degradation of MB were fitted to the first-order kinetics model of Equation (2).

(2) $$-\ln\frac{C_{t}}{C_{0}}=kt$$

where *C*_0_, *C_t_*, and *k* are the initial concentration of MB, the MB concentration at time t, and the rate constant, respectively.

## 3. Results and Discussion

### 3.1. Characterization of Materials

**Figure 1:**
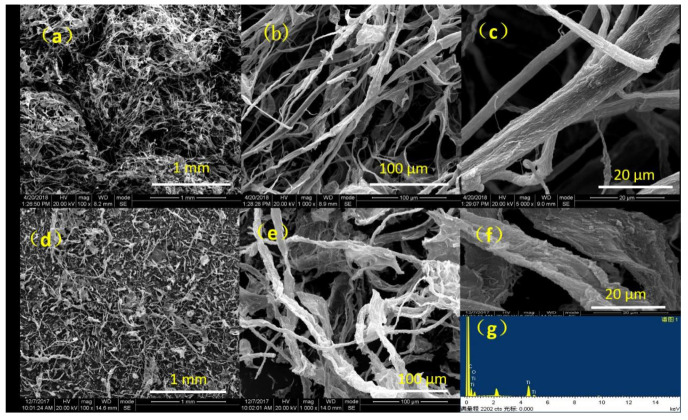
SEM images of carbon aerogels (CA) (**a**–**c**) and CA/TiO_2_ (**d**–**f**) and energy dispersive X-ray spectroscopy (EDX) results for CA/TiO_2_ (**g**).

Figure 1 shows the morphologies of CA/TiO_2_ and CA under different magnifications. Some significant differences can be observed. Compared with CA, the fibers in CA/TiO_2_ exhibited a rougher surface, and their average diameter increased from 7.05 to 8.9 μm. As expected, CA/TiO_2_ greatly retained the highly porous structure of CA. Figure 1e clearly displays the abundant pores in CA/TiO_2_. These pores could not only contribute to the adsorption of MB and strengthen the contact between MB and active sites, but also suppress the formation of boundaries and defects from carbon aggregation. Interestingly, the CA/TiO_2_ had a rather low density (18.5 mg/cm^3^), which was comparable to and even lower than those of some early reported carbon aerogels derived from biomass [38,39]. Additionally, the EDX results (Figure 1g) indicated that the three elements (C, O, and Ti) existed in the aerogel. Along with the SEM image in Figure 1f, it could be confirmed that TiO_2_ had been anchored on the carbon fiber scaffold to form a thin layer.

**Figure 2:**
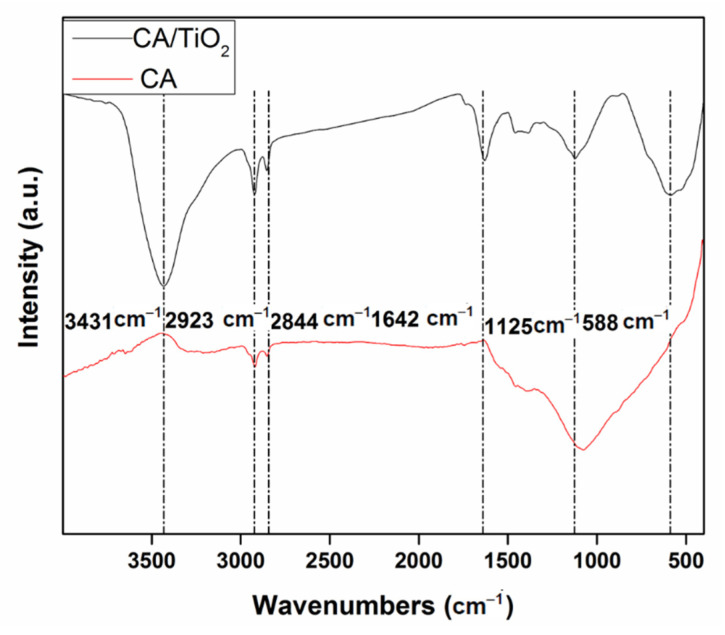
FTIR spectra of CA and CA/TiO_2_.

Figure 2 presents the FTIR spectra of CA/TiO_2_ and CA. The peaks at 2928 cm^−1^ and 1096 cm^−1^ are attributed to the C-H and C-O stretching vibrations, respectively. Compared with CA, CA/TiO_2_ displayed a predominant band at 3441 cm^−1^, which was assigned to the stretching vibration of surface water and hydroxyl groups on TiO_2_ [40,41]. It is worth noting that the hydroxyl groups could reduce the recombination possibilities of photo-carriers and generate active oxygen species during the photo-catalytic process [5,40]. Moreover, the new band for CA/TiO_2_ at 578 cm^−1^ was ascribed to the vibration of Ti-O-Ti, which consistently suggested that TiO_2_ had been incorporated in the carbon aerogel.

**Figure 3:**
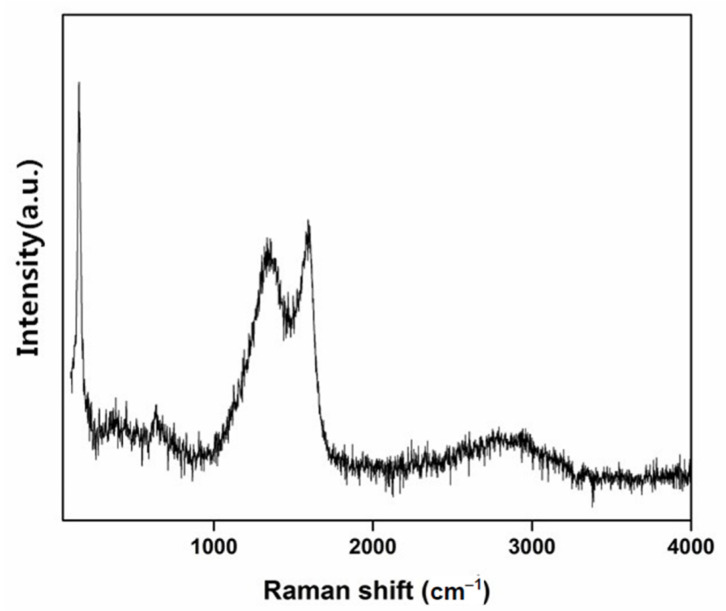
Raman spectrum of CA/TiO_2_.

A Raman spectrum was used to reveal the crystallographic characteristics of CA/TiO_2_. As shown in Figure 3, the peaks at 1584 and 1336 cm^−1^ were assigned to the D and G bands, respectively. The D band represents the sp^2^ units adjacent to structural defects, while the G band corresponds to sp^2^ planar and conjugated structures [42]. Furthermore, their intensity ratio of I_G_/I_D_ was employed to depict the order extent of carbon composites. In this study, the calculated I_G_/I_D_ value of CA/TiO_2_ was 1.02, smaller than that of CA without TiO_2_, as reported previously [37]. This implied the increased defects and lattice disorders of carbon in CA/TiO_2_ [43,44]. The band at 151 cm^−1^ represented the E_g_ model of anatase TiO_2_ [45]. Some early studies revealed that anatase TiO_2_ has higher photo-catalytic activity, which would benefit the photo-catalytic efficiency of CA/TiO_2_ [46].

**Figure 4:**
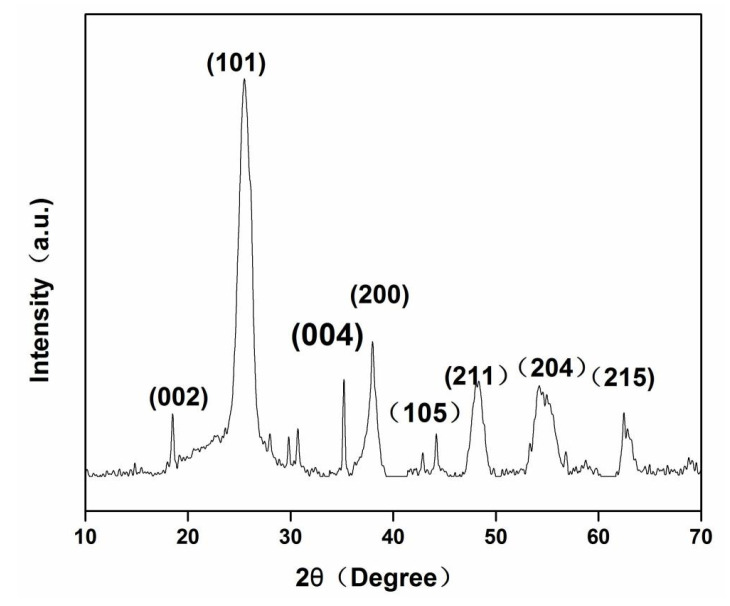
XRD pattern of CA/TiO_2_.

XRD was utilized to analyze the crystalline structures of CA/TiO_2_. As is well known, TiO_2_ has two main crystalline phases, anatase and rutile. Because of the high reactivity and chemical stability, most research interest has been focused on the anatase TiO_2_ [14]. However, anatase is a metastable polymorphic form and will transform to rutile upon heating, which hardly shows any photo-catalytic activity. For pure TiO_2_, this transformation roughly occurs at 730 °C, while this transition temperature varies according to the specific surface area, the particle size of TiO_2_, and its purity [47,48]. Furthermore, the presence of carbon phase could also affect the crystal growth of TiO_2_ in the corresponding composite [49,50]. Figure 4 depicts the XRD pattern of CA/TiO_2_. The strong peaks at 2θ = 25.5°, 35.3°, 38.0°, 4.1°, 54.1°, 55.1°, and 62.5° could be indexed to the (101), (004), (200), (105), (211), (204), and (215) crystal planes of anatase, respectively. It is interesting to notice that only anatase was observed in the XRD pattern. This seems contradictory to previous reports that when samples were carbonized at 800 °C, only rutile existed in the final products. It was believed that the interaction between the carbon phase and the anatase phase could have avoided the agglomeration and sintering of TiO_2_ particles and effectively stabilized the anatase phase, preventing the transformation from anatase to rutile, even at a high temperature [35].

**Figure 5:**
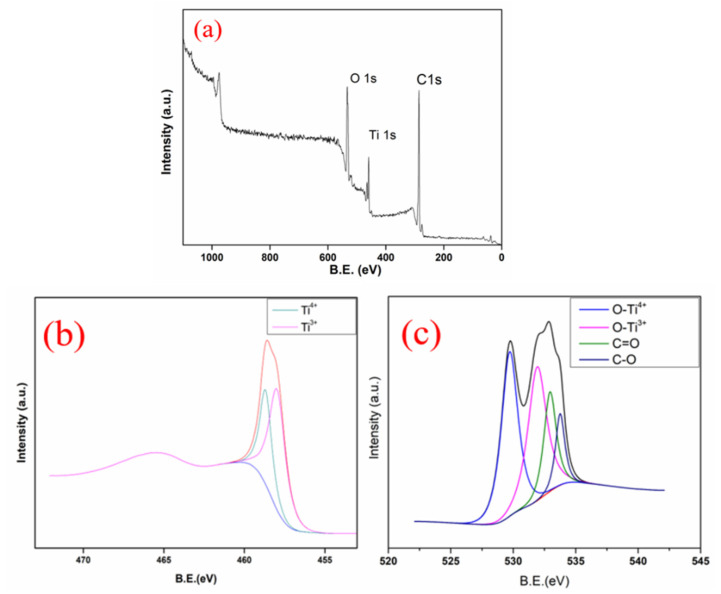
XPS spectrum (**a**) and deconvoluted Ti_2_p spectrum (**b**) and O1s spectrum (**c**) of CA/TiO_2_.

XPS (Figure 5a) was used to analyze the surface chemical composition of CA/TiO_2_. According to the literature, pure TiO_2_ presents only one peak of Ti^4+^, located at the binding energy BE = 459.1 eV in a deconvoluted Ti_2_p spectrum [51]. However, the deconvoluted Ti_2_p spectrum of CA/TiO_2_ (Figure 5b) displayed two components at 459.1 and 458.0 eV, respectively. The first peak corresponded to the Ti^4+^, while the other peak at 458.0 eV was attributed to the Ti^3+^ [52], suggesting that there was a reduction reaction of organic phase during carbonization [35,52].

In the XPS spectrum of O1s (Figure 5c), four peaks located at BE = 529.9, 531.6, 532.9, and 533.8 eV could be observed. As previously reported, the component at BE = 530.2 eV was assigned to the oxygen bonded to Ti^4+^, and the peak at BE = 531.3 eV corresponded to the oxygen bonded to Ti^3+^ [48]. The two components at 532.9 and 533.6 eV were attributed to the oxygenated surface groups of the carbon phase, like C=O and C-O. Notably, these oxygen-containing groups not only favored the dispersing and anchoring of TiO_2_ on the carbon scaffold, but also substituted for the lattice oxygen and formed Ti-O-C bonds, giving rise to carbon doping [53,54,55,56].

### 3.2. Optical Property

**Figure 6:**
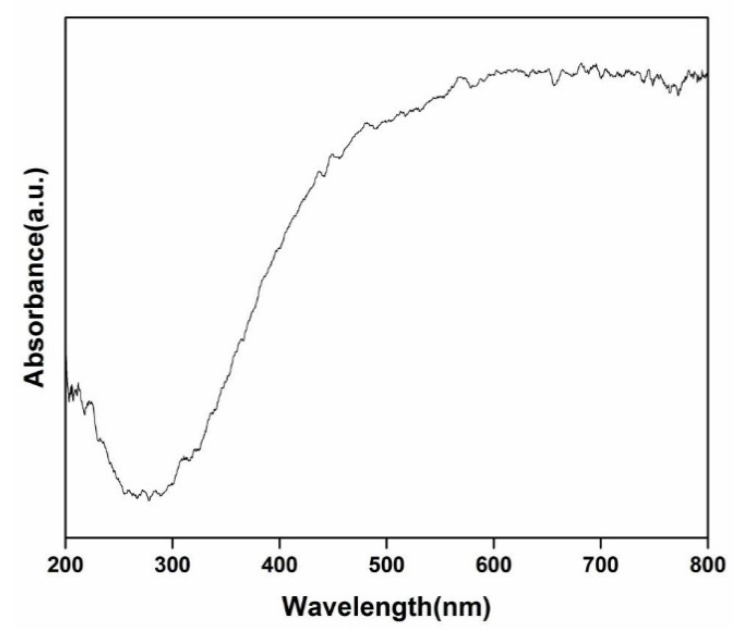
Diffuse reflectance spectrum of CA/TiO_2_.

Due to the intrinsic band gap, TiO_2_ could only absorb ultraviolet light. Figure 6 shows the diffuse reflectance UV–vis spectrum of CA/TiO_2_, which displayed a strong absorption in the visible light wavelength range of 400–800 nm. This result can be attributed to the following reasons. On the one hand, the quantum confinement by the well-dispersed TiO_2_ on the carbon scaffold and the carbon-doping as a result of the formation of Ti-O-C could remarkably narrow the band gap of TiO_2_ [54,57]. On the other hand, the oxygen vacancies generated during carbonization and the partially reduced Ti^4+^ as confirmed by XPS might have acted as a new state in between the band gap of TiO_2_, leading to the intense visible light absorption. This agrees well with a recent study on CNT/TiO_2_ for water splitting [58].

### 3.3. Adsorption and Photo-Catalytic Activity Study

**Figure 7:**
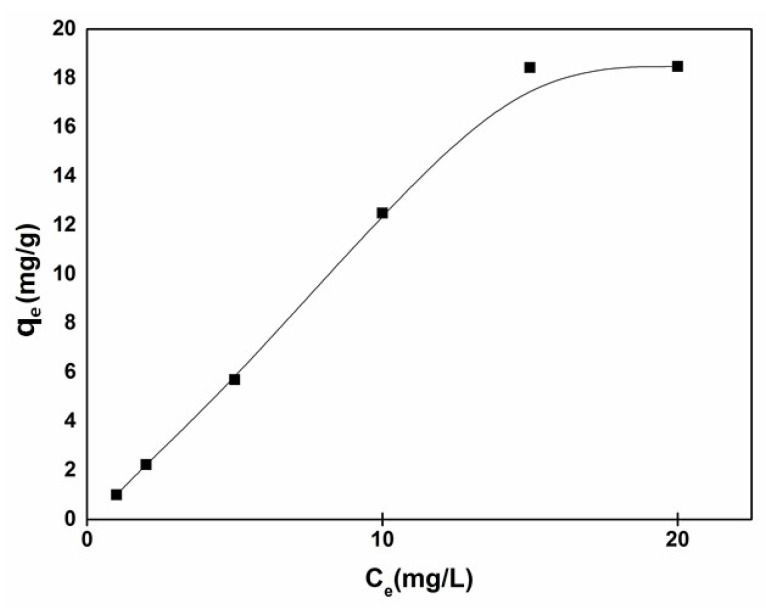
Adsorption isotherm of methylene blue (MB).

Figure 7 displays the MB adsorption curve of CA/TiO_2_. The adsorption capacity of CA/TiO_2_ for MB increased with the increase of MB concentration in the solution. Additionally, the maximum adsorption capacity was 18.5 mg/g, higher than those of many other similar materials. For example, Zhang [59] et al. prepared a TiO_2_/carbon@TiO_2_ composite with a core–shell structure; its adsorption capacity was 11.4 mg/g. Wang [54] prepared a TiO_2_/carbon composite using cotton as the carbon source, which exhibited an adsorption capacity of 8 mg/g.

The improved adsorption capacity of CA/TiO_2_ should be ascribed to the large amount of hydroxyl groups presented in CA/TiO_2_, which had a preferable affinity with the positively charged MB molecule. Additionally, the abundant interconnected pores favored the diffusing process of MB from the solution into the material. As a consequence, more MB molecules could be attracted around TiO_2_, giving rise to improved photo-catalytic efficiency.

**Figure 8:**
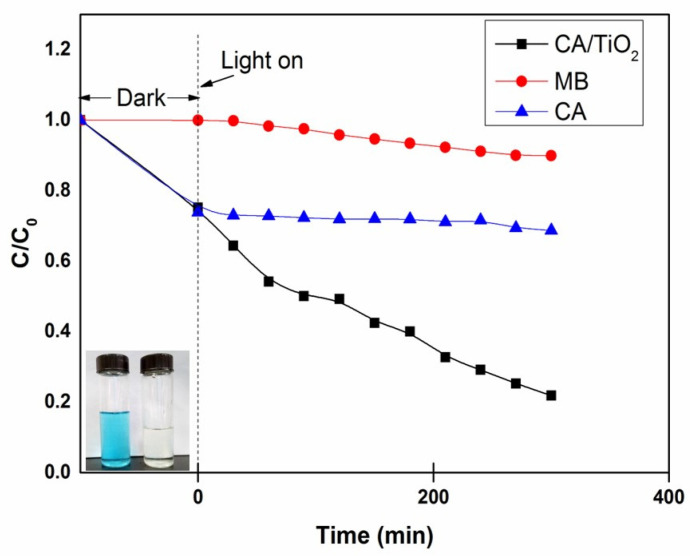
Photo-catalytic degradation of MB by CA/TiO_2_ and CA. The insert shows the color change of the solution before and after the CA/TiO_2_ treatment.

**Figure 9:**
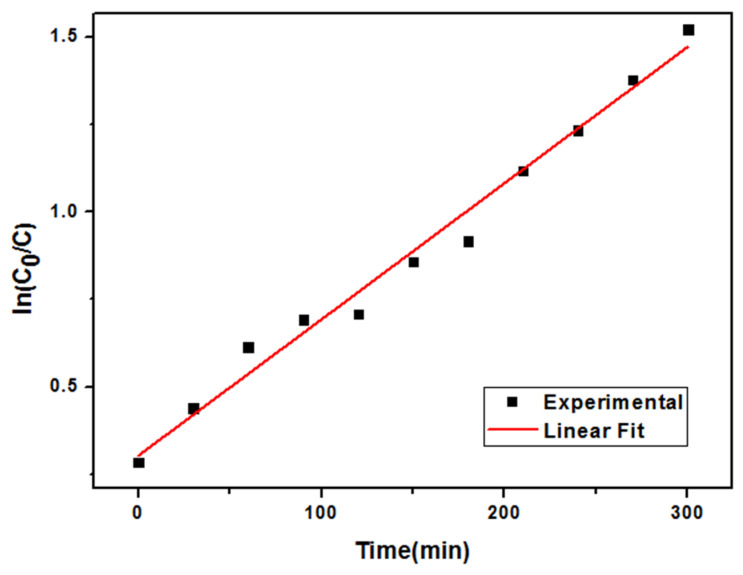
First-order degradation kinetics plot of MB by CA/TiO_2._

The photo-catalytic activity of CA/TiO_2_ was evaluated by monitoring the degradation of MB under a common incandescent lamp. Because the aerogel materials also had a strong adsorption towards MB, all samples were saturated with MB under dark conditions prior to the testing of photo-catalytic activity. Figure 8 compares the photo-catalytic performance of CA/TiO_2_ and CA. As expected, CA exhibited no photo-catalytic activity. By contrast, CA/TiO_2_ could efficiently degrade MB. Using only 15 mg of CA/TiO_2_, nearly 85% of MB in 50 mL solution with a concentration of 10 mg/L was degraded in 300 min, and the initial solution in blue was almost completely decolored after photo-degradation (see the inset image in Figure 8). In addition, the first-order degradation kinetics plot of MB by CA/TiO_2_ was analyzed and is shown in Figure 9 [54]. The plot appeared to have good linearity with a high correlation coefficient (R^2^ = 0.9828) and a *k* value of 0.0039 min^−1^. It is noteworthy that the photo-catalytic efficiency of CA/TiO_2_ was higher than those of many other similar photo-catalysts in the literature. For example, Esther [35] et al. prepared a carbon xerogel–TiO_2_ composite, and 800 mg of the photo-catalyst removed 90% of dye (800 mL, 10 mg/L) in 400 min. Wang [54] et al. used a TiO_2_–carbon fiber composite as the photo-catalyst to degrade MB under visible light. The results indicated that 120 mg composite could degrade 80% of the dye in 100 mL MB solution (10 mg/L) after 10 h. Chen [60] et al. synthesized a TiO_2_/carbon photo-catalyst to degrade MB under visible light, and 125 mg of the photo-catalyst degraded 90% MB in 250 mL solution (5 mg/L) after 7 h.

The superior photo-catalytic activity of CA/TiO_2_ could be explained by the following facts. The catalytic active site of TiO_2_ was anchored on the surface of carbon fiber to form a thin layer. This structure could reduce the distance that the photo-generated electrons and holes need to travel and decrease the recombination probability between these electrons and holes. Additionally, CA/TiO_2_ had a highly porous and interconnected three-dimensional framework structure, which could effectively prohibit the generation of boundaries and defects (which often lead to the recombination of photo-generated electron–hole pairs) due to carbon aggregation. Last but not least, the high adsorption capacity of CA/TiO_2_ resulted in the enrichment of MB around TiO_2_, which improved the contact between MB and the photo-catalyst and ultimately enhanced the photo-catalytic efficiency.

## 4. Conclusions

In this work, CA/TiO_2_ was synthesized via hydrothermal and carbonization processes using bamboo pulp fibers and Ti(OBu)_4_ as the raw materials. An instrumental analysis indicated that the catalytic active site of TiO_2_ was well dispersed and homogeneously anchored on the surface of carbon fibers. The obtained TiO_2_ had a crystalline structure indexed to anatase. This was resultant from the interaction between carbon and TiO_2_, which prevented the transformation from anatase to rutile during the high-temperature carbonization process. The obtained CA/TiO_2_ was highly porous and exhibited outstanding adsorption and photo-catalytic properties for MB decolorization. It is envisaged that the high-performance CA/TiO_2_ photo-catalyst from low-cost and sustainable bioresources may find great potential in treating organic dye polluted wastewaters.

## Data Availability

The data presented in this study are available within this article. Further inquiries may be directed to the authors.
